# Specificity and sensitivity of the social communication questionnaire lifetime screening tool for autism spectrum disorder in a UK CAMHS service

**DOI:** 10.1177/13591045221137196

**Published:** 2022-12-06

**Authors:** Amelia Staton, David Dawson, Nima Moghaddam, Barbara McGrath

**Affiliations:** 1 6123 University of Nottingham, UK; 2 4547 University of Lincoln, UK; 3 5314 Nottinghamshire Healthcare NHS Foundation Trust, UK

**Keywords:** Autism, social communication questionnaire, screening, CAMHS

## Abstract

**Introduction:**

The Social Communication Questionnaire is used to identify children and young people (CYP) who may require formal ASD assessment. However, there is a paucity of research on its utility in Children and Adolescent Mental Health Services. This evaluation aimed to determine the sensitivity and specificity of the Social Communication Questionnaire (SCQ) in a UK, Midlands CAMHS service.

**Method:**

Forty young people (mean age 13.75 years) were screened using the caregiver reported SCQ before completing ‘gold standard’ assessment.

**Results:**

The SCQ had a sensitivity of 80% and a specificity of 25.7%. ROC curve analysis indicated low diagnostic accuracy. Differences in predictive accuracy of SCQ and diagnostic standard were statistically significant (*p* < 0.0001).

**Conclusion:**

This evaluation builds on previous research suggesting that the SCQ may not be an efficient screening tool in CAMHS settings.

## Introduction

Whilst an estimated 1.76% of school age children in the UK meet the diagnostic threshold for ASD (Roman-Urrestarazu et al., 2021), autism presentations appear to be significantly higher in looked after children and children who have experienced attachment trauma (Rutter et al., 1999). These findings are consistently replicated in several cross-cultural studies (Green et al., 2016; Hoksbergen et al., 2005; Meltzer et al., 2003; Sadiq et al., 2012), providing support within the wider literature around overlapping autism and attachment symptomologies (McKenzie & Dallos, 2017; Minnis et al., 2020). Clinical observation has highlighted several similarities in these presentations such as inflexibility, atypical play, emotional dysregulation, sensory needs and communication difficulties (Moran, 2010). In addition to overlapping symptom profiles, the literature suggests that autistic children may be more likely to develop insecure attachments (Rutgers et al., 2004; Naber et al., 2007). This may be exacerbated by systemic factors such as parental mental health (Berry & Drake, 2010) and parent’s own attachment experiences (McKenzie & Dallos, 2017). This is likely to impact on psychological well-being, with recent meta-analysis indicating that mental health difficulties are significantly higher in autism populations (Lai et al., 2019), and thus may be overrepresented in mental health settings.

Whilst there appears to be a complex relationship between these presentations, their differentiation is crucial in minimising the risk of misdiagnosis. Screening tools are used to identify children and young people (CYP) who may require further investigation for a possible diagnosis of ASD. The Social Communication Questionnaire (SCQ; Rutter, Bailey, & Lord, 2003) is one such tool commonly used in clinical practice for children aged 4 years and older. Developed from the Autism Diagnostic Interview – Revised (ADI-R; Rutter, Le Couteur, & Lord, 2003; Lord et al., 1994), the SCQ is a 40-item caregiver-reported questionnaire that explores language development and social communication throughout the child’s developmental history (SCQ Lifetime) and behaviours observed in the past 3 months (SCQ Current). When evaluating the accuracy of screening tools and diagnostic tests, it is essential to consider whether the test can correctly identify individuals with a condition (sensitivity) and correctly classify those who do not (specificity). This is of particular importance in clinical settings where standardised screening and assessment tools are used to provide diagnoses and determine appropriate intervention. When assessing the diagnostic validity in clinical samples, the SCQ was found to have a sensitivity (*Se*) of 0.96, specificity (*Sp*) of 0.80 and an area under the curve (AUC) of 0.95 indicating high diagnostic accuracy (Berument et al., 1999). Recent meta-analysis examining the utility of the SCQ found the tool had moderate diagnostic accuracy (pooled AUC of 0.82) and thus could be considered an acceptable screening measure (Chesnut et al., 2017). Though, these findings have not been consistently replicated with several studies highlighting lower diagnostic accuracy when used with younger children (Allen et al., 2007; Eaves, Wingert, Ho, & Mickelson, 2006, 2007; Marvin et al., 2017; Norris & Lecavalier, 2010) and children with developmental and intellectual disabilities (Allen et al., 2007; Corsello et al., 2007; Snow and Lecavalier, 2008). Divergence in findings between studies is hypothesised to be a result of using different forms of the SCQ (Chesnut et al., 2017; Wei et al., 2015), the age of sample (Barnard‐Brak et al., 2016), and varying sample populations (Chesnut et al., 2017). Furthermore, only one SCQ validation study included individuals with co-existing anxiety/mood disorders within the larger sample (Corsello et al., 2007), and two referenced inclusions of CYP with ‘behavioural disorders’ (Allen et al., 2007; Norris & Lecavalier, 2010). Whilst the interrelation between autism, attachment and mental health difficulties have been highlighted in the literature, this appears to be rarely considered when developing and validating screening tools.

The overlap between autism, attachment and co-occurring mental health presentations has considerable implications on assessment and diagnosis. Screening tools that are not effective in distinguishing autism from other presenting difficulties may lead to inappropriate clinical and educational intervention. In addition, tools with poor diagnostic accuracy increase the likelihood of unnecessary assessments and burden on CYP and their families. During a time of increasing demand and rising pressure within specialist CAMH services (Ludlow et al., 2020), efficient assessment pathways and effective use of service resources are essential. Hollocks et al. (2019) evaluated the utility of both the SCQ Lifetime and Current forms in this setting. The results suggested low diagnostic accuracy in young people with co-existing mental health needs; SCQ Lifetime (*Se* 0.87, *Sp* 0.12, AUC 0.52), SCQ Current (*Se* 0.72, *Sp* 0.35, AUC 0.56). However, the evaluation compared the SCQ using only the Autism Diagnostic Observation Schedule, Second Edition (ADOS-2; Hus & Lord, 2014) as the diagnostic standard. Current ‘gold standard’ assessment includes use of both the ADOS-2 and the Autism Diagnostic Interview – Revised (Lord et al., 1994) as this has been found to have the most reliable results (Risi et al., 2006).

Therefore, this study aimed to partially replicate Hollocks et al. (2019) evaluation of the utility of the SCQ in a CAMHS service, using the ‘gold standard’ assessment (ADOS-2 and ADI-R) as the diagnostic standard.

## Service context

**Figure 1.**
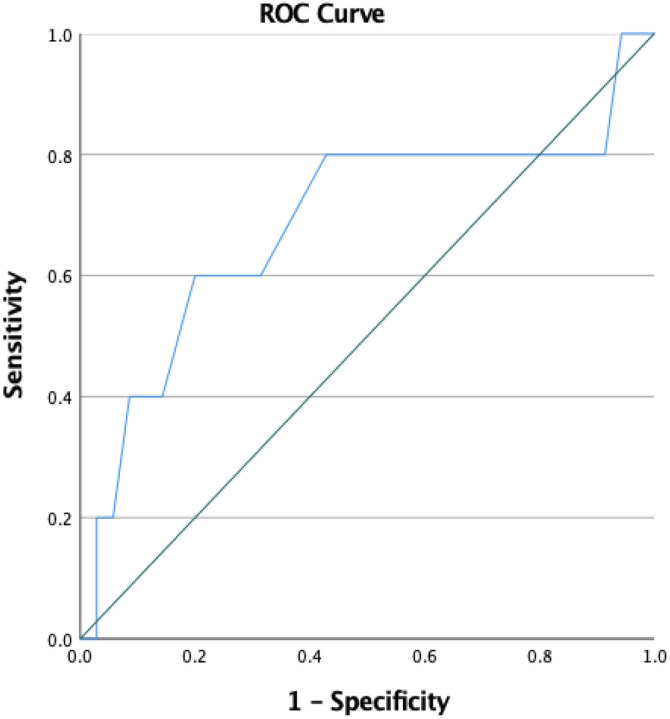
ROC curve analysis.

This evaluation was conducted in a UK Midlands, CAMH service. The service provides assessment and diagnosis of autism for young people (<18 years) currently receiving input from Community CAMHS provision. The ASD assessment clinic was established in 2009 due to increased wait-list times in ASD specialist services. The clinic is comprised of 3 × Clinical Psychologists (0.6 wte), 1 x Specialist Practitioner (0.2 wte) and 1 × Independent Non-Medical Prescriber (0.2 wte). The team meet fortnightly and all meetings are multi-disciplinary, involving members from at least two professional backgrounds (Figure 1).

## Aims

1. To evaluate the sensitivity and specificity of the SCQ screening tool in this CAMH service.

## Method

All referred young people between June 2017 and January 2022 were included if they had a complete assessment at the time of data collection. No other exclusion criteria were applied. Once referred to the ASD assessment clinic, the caregiver completed SCQ and referral information were reviewed by the MDT and a decision was made on whether there was a rationale for further assessment. The assessment process involved the Autism Diagnostic Observation Schedule (ADOS; Hus & Lord, 2014) and the Autism Diagnostic Interview – Revised (ADI-R; Rutter, Le Couteur, & Lord, 2003).

This service evaluation was registered with the Trust Research and Evidence department in line with the Health Research Authority (HRA) guidelines. The data was routinely collected by the service and was anonymised prior to analysis (Tables 1 and 2).

**Table 1.** SCQ cut-off and diagnostic outcome.

	ASD	No diagnosis of ASD
SCQ ≥ 15	4	26
SCQ < 15	1	9

**Table 2.** Sensitivity and specificity of SCQ screening tool.

	*n*	Sensitivity	Specificity	PPV^1^	NPV^2^	McNemar’s test	Odds ratio χ^2^	Youden’s J
SCQ ≥ 15	40	80%	25.7%	13.3%	90%	*p* < 0.0001	21.3	0.06

^1^Positive predictive value.

^2^Negative predictive value.

## Measures

### SCQ – lifetime

The SCQ Lifetime (Rutter, Bailey, & Lord, 2003) is a parent/caregiver completed ASD screening tool developed for individuals over the age of four. It includes 40-items exploring language development and social communication. The SCQ Lifetime form asks respondents questions that span the child’s developmental history (i.e., *“can you have a to and fro conversation with her/him that involves taking turns or building on what you have said?”*) and a specific period of time (i.e., *“when she/he was aged 4 to 5, did she/he show a normal range of facial expressions?”*). Scores can range from 0 to 40 with a suggested clinical cut-off of 15 and above indicating the need for ASD assessment.

### ADOS-2

The Autism Diagnostic Observation Schedule (ADOS) is a standardised assessment that covers several domains: communication, social interaction, and play (Hus & Lord, 2014). It is designed to be used by clinicians to assess current behaviour and can be used with children of varying ages and developmental stages. In line with best practice, this assessment is recorded and reviewed in a multi-disciplinary context. Recent meta-analysis found sensitivity ranged between 0.89 and 0.92 and specificity between 0.81 and 0.85 indicating high diagnostic accuracy (Lebersfeld et al., 2021).

### ADI-R

The Autism Diagnostic Interview – Revised (Rutter, Le Couteur, & Lord, 2003) is a structured interview completed with the young person’s parent/caregiver that covers “reciprocal social interaction, communication and language, interests and behaviours”. It is designed to be used in conjunction with the ADOS-2. Findings from meta-analysis have found a pooled sensitivity of 0.75 and specificity of 0.82 indicating high diagnostic accuracy (Lebersfeld et al., 2021).

## Statistical analyses

To assess the predictive accuracy of the SCQ, we planned to use McNemar’s analysis (McNemar, 1947). Given that we included all service referrals with complete assessment data, a post-hoc power analysis was completed. Power calculations were completed using G*Power version 3.1 (Faul et al., 2009). Statistical analysis was performed using SPSS version 27. The sensitivity and specificity were calculated for the SCQ, with the ADOS and ADI-R as the diagnostic standard. McNemar’s test was used to compare the binary response for these matched pairs data, represented as a 2 × 2 contingency table. This table showed the number of observed outcomes in each category (McNemar, 1947). Positive predictive value (the likelihood that if you score above the SCQ clinical cut-off that you will receive a diagnosis of ASD) and negative predictive value (likelihood that if you score below the SCQ clinical cut-off that you will not receive an ASD diagnosis) were calculated using suggested formulas (Molinaro, 2015). A Receiver Operating Characteristic (ROC) curve analysis was completed to determine the area under the curve (AUC) and to identify optimal cut off for diagnostic performance. We used widely agreed criteria to interpret the area under the curve (AUC); low (<0.7), moderate (0.7–0.9) or high accuracy (>0.9) (Hanley & McNeil, 1982; Swets, 1988). Youden’s index was calculated (sensitivity + specificity) – 1) to evaluate discriminative power of the SCQ (Youden, 1950). A Youden’s index of 0.6 or above is considered ‘acceptable’ for diagnostic tests (Chen et al., 2015).

## Results

Between June 2017 and January 2022, 121 referrals were screened using the SCQ with 60 (49.5%) of these accepted for assessment. 20 cases were excluded from analysis due to incomplete assessment: did not opt in for assessment (*n* = 1); declined assessment (*n* = 1); assessment postponed on family’s request (*n* = 2); ongoing assessment (*n* = 7); discharged due to change in presentation (*n* = 1); aged out of service whilst on waiting list (*n* = 2), and missing data (*n* = 6). 10 cases scored below the SCQ suggested clinical cut off but were recommended for assessment following a multi-disciplinary discussion.

The final sample consisted of 40 young people (62.5% female, 37.5% male), mean age of 13.75 (range = 9–17 years; *SD* = 2.15; *SE* = .341). A post hoc analysis was performed to calculate power using obtained sample size, odds ratio and α level (α = .05). Results indicated an achieved power of 98% with a significance criterion of (α = .019).

Five of the 40 cases (12.5%) were assessed (using the ADOS and ADI-R) as meeting diagnostic criteria for ASD. The remaining 35 young people (87.5%) were deemed to have “significant emotional/psychological difficulties” (e.g., anxiety, low mood, developmental trauma, and attachment difficulties) that required further assessment and intervention with their usual care team. Of the five who met criteria for diagnosis of ASD, 3 identified as female and two identified as male. Due to the nature of the service and referral pathway, all young people were referred by a CAMHS professional currently involved in their care.

## Social communication questionnaire

The overall sample had a mean score of 20.38 (range 1–35; *SD* = 7.72). 75% of the sample met the SCQ clinical cut-off (≥15) with a mean score of 23.20 (range 15–35; *SD =* 5.83). 15.3% of those that met the suggested clinical cut-off received a diagnosis of ASD. 25% of the sample scored below the clinical cut-off (<15) with a mean score of 10.22 (range 1–14; *SD* = 3.96). One (11.1%) of the cases scoring below the SCQ cut off met diagnostic criteria for ASD following assessment (Table 1).

## Sensitivity and specificity of the SCQ in-CAMHS population

Using the tools recommended clinical cut-off (≥15), the SCQ had a sensitivity of 80% and a specificity of 25.7%. The negative predictive value was 90% and the positive predictive value was 13.3%. McNemar within-subjects chi-squared χ^2^ test (McNemar, 1947) was used to assess whether differences in the predictive accuracy of the SCQ tool and diagnostic standard (ADOS and ADI-R) were statistically significant. The chi-square statistic and odds ratio (χ^2^ = 21.3) indicated that the odds of positive classification by the SCQ were 21.3 times greater than the odds of positive classification by the diagnostic standard (ADOS and ADI-R) (*p* < 0.0001) (Table 2).

For the total SCQ score, the AUC was 0.68 (95% CI 0.38–0.98) with a Youden’s index of 0.06 indicating low diagnostic accuracy. The findings suggest that 74.3% of those that score above the SCQ cut-off will be assessed to not meet the diagnostic threshold. In addition, at least one in five young people with ASD will score below the SCQ cut off. ROC curve and Youden index analysis indicated that the optimal cut off for diagnostic performance in this setting would be ≥21, maximising Youden’s J at 0.37, retaining sensitivity at 80% and increasing specificity to 57% (Figure 1).

## Discussion

The aim of this evaluation was to evaluate the sensitivity and specificity of the SCQ screening tool in this setting. This evaluation arose as a result of the growing literature around overlapping attachment and autism symptomologies and the implications of this on screening and assessment, particularly in CAMH services. Consistent with previous evaluation of another service (Hollocks et al., 2019), our findings suggest that the Social Communication Questionnaire (SCQ) may not be an efficient screening tool in this clinical context.

With a low diagnostic accuracy and an estimated sensitivity of 80%, at least one in five young people who meet the diagnostic criteria for ASD will be screened out prior to assessment. In addition, almost three quarters of those scoring above clinical cut off do not receive a diagnosis of ASD when formally assessed. The differences in the predictive accuracy of the SCQ tool and diagnostic standard (ADOS and ADI-R) were statistically significant. In comparison to other studies (Berument et al., 1999; Chesnut et al., 2017), the sensitivity, specificity, and diagnostic accuracy of the SCQ in this service is considerably lower than expected. The differences in study samples may explain the divergence in findings between this evaluation and previous research. The findings of our evaluation appear consistent with previous evaluation of the SCQ Lifetime form in an East England CAMHS service which found the SCQ to have low diagnostic accuracy and poor specificity (Hollocks et al., 2019). These findings have considerable implications on clinical practice with young people likely to be excluded from appropriate clinical and educational intervention due to inaccurate diagnosis. The low specificity of this tool increases the risk of unnecessary and burdensome assessments on young people and their families. In addition, screening tools with a likelihood of high false positives increase clinician workload and clinic waiting times. At a time of unprecedented waiting times and staff shortages within specialist CAMHS, it is essential that limited resources are used efficiently (Ludlow et al., 2020).

The SCQs limited ability to accurately screen out those that are unlikely to require further ASD assessment may be due to the complexity of cases in CAMHS. Although research on the incidence of attachment presentations in CAMH services is limited, insecure attachment styles are often correlated with mental health difficulties (Goodwin, 2003) and thus may be overrepresented in these settings. Furthermore, attachment difficulties are correlated with emotional dysregulation, heightened threat response and issues around interpersonal functioning (Mikulincer & Shaver, 2012); characteristics which arguably underpin an array of mental health presentations referred into CAMHS. When considering the utility of ASD screening tools in mental health settings, it could it postulated that the SCQ is inadequate in differentiating between attachment difficulties, autism and co-occurring mental health difficulties. For example, items on the SCQ Lifetime form such as *“does her/his facial expressions seem appropriate to the particular situation, as far as you can tell?”,* could be answered *“no”* for children with autism or attachment difficulties. Research evaluating differences in facial expression have found that individuals with autism (Trevisan et al., 2018) and insecure attachment (Altmann et al., 2021) have atypical facial expressions compared to their counterparts. Similarly, other questions on the SCQ such as items pertaining to communication of empathy (e.g., *“when she/he was 4 to 5, did she/he ever try to comfort you if you were sad or hurt?”*) may also lack differentiation as difficulty communicating empathy may be observed in both autism and attachment presentations (Davidson et al., 2015). Despite this overlay in symptomologies (Lai et al., 2019; McKenzie & Dallos, 2017; Minnis et al., 2020), attachment and mental health presentations appear to be rarely considered when developing ASD screening tools.

As the SCQ is not designed to provide formal diagnosis, prioritising sensitivity over specificity in this context is sensible. However, the SCQs extremely limited ability to screen out young people in which autism is not present, indicates that it is not an appropriate screening tool in this service. ROC analysis and Youden’s index suggests an optimal cut-off of ≥21 for diagnostic performance in this setting, which would reduce the number of false positive cases and therefore reduce the number of unnecessary assessments. However, this clinical threshold would not increase sensitivity and therefore would not decrease the number of false negative cases. As discussed in Hollocks et al. (2019) evaluation, there are alternative age-appropriate ASD screening tools. For example, the Social and Communication Disorders Checklist (SCDC; Skuse et al., 2005), the Social Responsiveness Scale (SRS; Constantino et al., 2003), and the Children’s Communication Checklist (CCC; Bishop, 1998). Though, the SCQ has been found to out-perform these screening tools (Chandler et al., 2007) and therefore using an alternative tool is unlikely to address concerns regarding screening accuracy in this clinical context. In addition, systematic review of ASD screening tools highlighted that only the SCQ and SRS had been examined in more than two studies (Hirota et al., 2018). Thus, the wider literature does not appear to lend support for an alternative ASD screening tool in this clinical context. The development and validation of a novel measure designed to distinguish between overlapping autism, attachment, and mental health symptom profiles is urgently required.

## Recommendations for clinical practice

To address these issues, it may be appropriate to include additional screening tools alongside the SCQ to support clinical judgement and decision making. Mental health screening measures such as the Revised Children’s Anxiety and Depression Scale (RCADS; Chorpita et al., 2005) and the Strength and Difficulties Questionnaire (SDQ; Muris et al., 2003) are already routinely collected in CAMHS and provide a broader understanding of the child’s presentation. However, it may be useful to consider the results of these measures during ASD clinic MDT discussions to aid formulation of the young person’s presenting difficulties. Collecting information from multiple sources (i.e., teachers, GPs) is also recommended during the screening and assessment process (National Institute for Health and Care Excellence [NICE], 2017).

Unlike broader psychological measures, the evidence base for attachment screening tools and assessments is limited. Whilst there are interviews and measures such as the Child Attachment Interview (CAI; Target et al., 2003) and the Inventory of Parent and Peer Attachment (IPPA; Gullone & Robinson, 2005), a systematic review concluded that these should be used with caution in clinical practice due to low inter-rater reliability (Jewell et al., 2019). In addition, these tools are unlikely to support differentiation between autism and attachment presentations. The Coventry Grid (Moran, 2010) was developed as a result of clinical observations that identified similarities in autism and attachment symptom profiles. Although the tool aimed to support clinicians to recognise the differences in presentations, it has faced criticism for conflating different attachment styles (McKenzie & Dallos, 2017). However, used judisicously, the Coventry Grid may support clinicians in the ASD assessment clinic during the screening and assessment process.

## Limitations

The results of this evaluation must be considered in the context of the methodological limitations. Due to the nature of this service specific evaluation, only a small sample of young people were included. Although the sample was representative of the types of cases seen in this service, the conclusions drawn from this study may not be generalisable. However, given the consistency of our findings with Hollocks et al. (2019) study, it could be argued that the limitations of the SCQ may extend to other CAMH services. Whilst the use of post hoc analysis to calculate power indicated an achieved power of 98% with a significance criterion of (α = .019), research using Monte Carlo simulation suggests that this type of power analysis may be inaccurate (Zhang et al., 2019). However, due to the nature of this evaluation a priori power analysis to calculate the required sample size was not possible.

The sensitivity of the SCQ in this service has been estimated based on the available data. However, this data is likely to be incomplete as only cases with full assessments were included in analysis. Whilst some cases scoring below the clinical threshold went on to be assessed due to clinical judgement, other cases scoring below this threshold were screened out prior to assessment. It is unclear whether there were false negatives among these screened out on the basis of SCQ scores and thus sensitivity and negative predictive value could indeed be lower. Additional research is needed determine true sensitivity of the SCQ in this setting. To calculate the true negative predictive value, a random sample of referrals would need to be formally assessed irrespective of the SCQ screening score. However, it is acknowledged that NHS CAMH services are overstretched and under resourced which poses significant challenges to completing research in these settings.

## Conclusions

This evaluation builds on the findings and subsequent recommendations from Hollocks et al. (2019) study suggesting that the SCQ may not be an efficient screening tool in CAMHS settings. Further research is needed to develop a novel ASD screening measure for use in CAMHS settings, with a particular focus on distinguishing between autism, attachment, and co-occurring mental health presentations. For current clinical practice, it is recommended that screening processes consider multiple sources of information and include judicious use of available measures and tools to support clinical judgement.
